# Studies on surface modification of polypropylene composite bipolar plates using an electroless deposition technique

**DOI:** 10.1039/d0ra00461h

**Published:** 2020-06-25

**Authors:** Rungsima Yeetsorn, Walaiporn Prissanaroon Ouajai, Kannika Onyu

**Affiliations:** Department of Industrial Chemistry, Faculty of Applied Science, King Mongkut's University of Technology North Bangkok Bangkok 10800 Thailand rungsima.y@sci.kmutnb.ac.th; Thai-French Innovation Institute, King Mongkut's University of Technology North Bangkok Thailand

## Abstract

A direct methanol fuel cell (DMFC) is predominantly noticeable because it can convert chemical energy directly into electrical energy with higher energy conversion efficiency (∼65%) compared to the efficiency of traditional combustion engines (40%) and with lower emissions. Henceforth, it is one of the new electrical generators that is becoming an important source of cleaner power in modern life. One of the key obstacles in designing and assembling the DMFC is contact resistance between interfaces of fuel cell components. A major source of the contact resistance in the DMFC arises from the contact between gas diffusion layers (GDLs) and the bipolar plates (BPs). A poor interface contact decreases the actual contact area, leading to an electrical voltage drop across these interfaces. Decreasing surface resistivity of BPs is one of the major approaches to reduce contact resistance in fuel cells. Present-day methods use a polypropylene composite as BPs to replace metallic or graphite BPs to reduce the overall weight of the DMFC stack. Unfortunately, polymeric composites typically provide higher surface resistance than the other BPs do. Coating copper on polypropylene composite plates was strategically manipulated by an electroless deposition (ELD) technique to decrease surface resistance. The coating process consists of pretreatment, adhesion improvement, and electroless deposition. Prior to ELD, the surfaces of the composite plates were treated by plasma treatment and then silanization was conducted using *N*-3-(trimetylpropylsilyl)diethylenetriamine (TMS) to improve adhesion. Palladium(ii) chloride (PdCl_2_) was used as a catalyst for the ELD process. Successful modification of the surfaces was confirmed by morphology investigation *via* scanning electron microscopy, diagnoses of chemical surface characteristics using ATR-Fourier-transform infrared spectroscopy (ATR-FTIR) and X-ray photoelectron spectroscopy (XPS), physical surface characterizations with a contact angle measurement, electrical conductivity measurements, and surface adhesion test, while also observing corrosion behavior. In order to complete a viability study of using modified copper-coated BP for the DMFC, an *in situ* cell performance test was conducted. The results of the experiments pave the way for a feasible modification of the BP surfaces to be considered as suitable BPs for usage in fuel cells.

## Abstract 2

The DMFC is predominantly noticeable because it can convert chemical energy directly into electrical energy with higher energy conversion efficiency (∼65%) compared to the efficiency of traditional combustion engines (40%) and with lower emissions.

## Graphical abstract

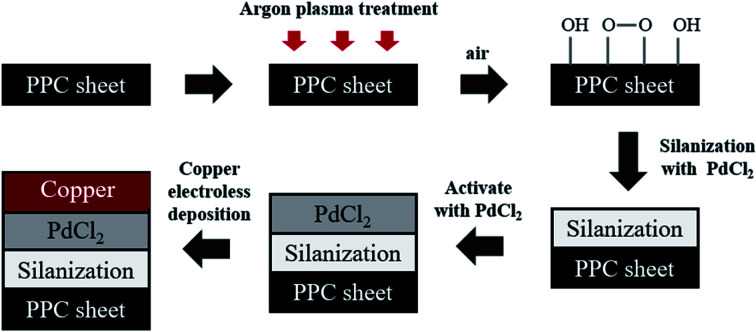

## 1. Introduction

The DMFC is a subcategory of polymer electrolyte membrane fuel cells, in which methanol is supplied as fuel. The main advantages of the DMFC are the ease of transport of alcohol, and reasonable energy density of a stable liquid at a wide range of environmental conditions. Therefore, it can be well operated under low operating temperatures (from room temperature up to 80 °C). DMFCs are primarily targeted for portable applications, where energy and power density are more important than efficiency.^1^ DMFC performance is theoretically determined by concerning losses of cell voltage as indicated in the following equations.^2,3^

1 *E*_cell_ = *E*° − *η*_act_ − *η*_ohmic,m_ − *η*_ohmic,BP_ − *η*_ohmic,GDL_ − *η*_conc_

where *η*_act_ represents the activation loss which is the voltage loss associated with electrochemical reactions occuring in the cathode and anode sides, and *η*_conc_ is the concentration loss depending on limitation of mass transfer. The ohmic loss or resistive loss, due to resistance of the membrane, BPs, and gas diffusion layers, are defined by *η*_ohm,m_, *η*_ohm,BP_ and *η*_ohm,GDL_ respectively. According to eqn (1), the ohmic loss mainly occurs because of material resistance (eqn (2)) including the resistance to flow of electrons through materials (*R*_electronics_), the resistance to flow of ions in a membrane electrolyte (*R*_ionic_), and contact resistance between cell components (*R*_contact_).^4^

2 *R*_materials_ = *R*_electronics_ + *R*_ionic_ + *R*_contact_

Many factors affect the contact resistance such as surface morphology of cell components, contact pressure at the material interfaces, the electrical conductivity of components, corrosion resistance of surface coating, corrosion resistance of gas diffusion layer and bipolar plate,^2^ clamping force, and surface roughness.^5^ In terms of surface morphology of GDLs and BPs, their surfaces are required to be as smooth as possible in order to enhance contact areas between them. Polishing BP surface is a choice for consideration to reduce the surface roughness, there is a case study on polishing commercial BPs (BMC 940-8649 flat composite plaques).^6^ When the plaques were polished by grit-600 sandpaper with thickness reduction from 1.00 μm to 0.76 μm, the contact resistance of BMC decreased from 32 mΩ cm^2^ to 24 mΩ cm^2^. Increasing surface electrical conductivity and corrosion resistance is another interesting approach for decreasing the contact resistance. Noble metals such as gold,^7,8^ Ni,^9–11^ and Zr^12^ were utilized as a coating material to achieve set target contact resistance. The simplest solution is to apply high clamping pressure during a cell or stack assembling process. The clamping pressure will squeeze the GDLs and BPs together, thus eliminating gaps between their interfaces. While utilizing this pressure method, carbon fibers in the GDL structure can penetrate the surface of composite bipolar plates causing the formation of conductive pathways.^13^ However, when clamping is performed with too much force, it can result in mechanical failure of the cell components in long term use, which directly influences fuel cell performance. The material electrical conductivity, either surface conductivity or volume conductivity, is mandatory factor to consider in order to reduce the ohmic losses and contact resistance. As known, contact resistance refers to the capability of electron transfer through surface components in fuel cells, so the surface conductivity significantly impacts on the contact resistance. Although the surface morphology and contact pressure are appropriate, the low surface electrical conductivity leads to a high contact resistance. This research article addressed on improving surface electrical conductivity of polymeric composite BPs. A light-weight composite BP made from polypropylene composite was highlighted in this research, since the use of these composites is relevant to the reduction of overall DMFC weight and the BP productivity enhancement (*via* an injection molding process). Nevertheless, the electrical conductivity of the polypropylene composite is inferior to that of a metal and graphite. The lower surface conductivity of the composite mainly comes from a polymer-rich layer formed during the injection in a molding process.^14,15^ The study literature mentioned above brought about an idea of fixing this weakness by coating a high conductive metal on the polypropylene composite BPs. To achieve a successful coating, two main procedures: surface treatment, an electrically conductive coating, must be performed.^8^ The surface treatment, such as sandpaper scratching,^16^ chemical etching, or plasma treating,^17^ is used to get rid of the polymer-rich layer on the surface of the polymer composite. The electrically conductive coating is the major step in laying conductive material; graphite, ZrN, TiN, Ni–Mo–P, Cu, Ni, Au, and graphene-coated copper plate^8,18–20^ for instance, on another material substrate. In general, coating conductive materials on metallic BP surface is intended to prevent corrosion, while surface conductivity can be maintained.^18^ Coating surface of polymer composite BPs is usually aimed at improving their surface electrical conductivity, however; it is quite difficult to achieve good interfacial adhesion between a coating material and a polymeric substrate by reason of the different polarity of surfaces. Copper has been applied as a material for fuel cell components, for example, copper-inserted BPs,^21^ multilayer-coated monoplates,^8^ and copper current collectors because of its high electrical conductivity (5.85 × 10^4^ S cm^−1^).^22^ In case of multilayer-coated polymeric monoplates, copper was firstly coated on a polymer monoplate (polycarbonate), and the copper layers were covered by nickel and gold layers, respectively.^8^ The copper was selected to be the first coated layer, since it provided the strongest interfacial interaction between the polymer substrate and the coating layer, compared to other two metals. Moreover, the coating copper on a polymer plate possesses strong coating layer, a simple process, and inexpensive cost. Some restrictions of multilayer coating on composite plates are surface fracture and metallic layer peeling off. The copper coating phenomena should be investigated, nevertheless, coating metals on polypropylene composite BPs has not been reported extensively. This work was a preliminary work to study the copper coating on polypropylene composite BPs *via* an electroless deposition technique before stepping to multilayer coating or using other noble metals for coating composite BPs in the future work. Susceptibility to corrosion must be taken into consideration since hydrogen ions are produced in the mechanism of redox reaction resulting in a pH of 2–3 ideal conditions for the occurrence of material corrosion. An application of applying copper-coated plastic for a flexible pH sensor used in acidic circumstance was published,^23^ thus our research team would like to observe the use of copper-coated polypropylene composite BPs in the DMFC operation condition. There are several techniques^24^ for coating copper on a substrate, for example, adhesion with glue, sputtering, vacuum coating, spray coating, electroplating and electroless plating.^25^

**Fig. 1:**
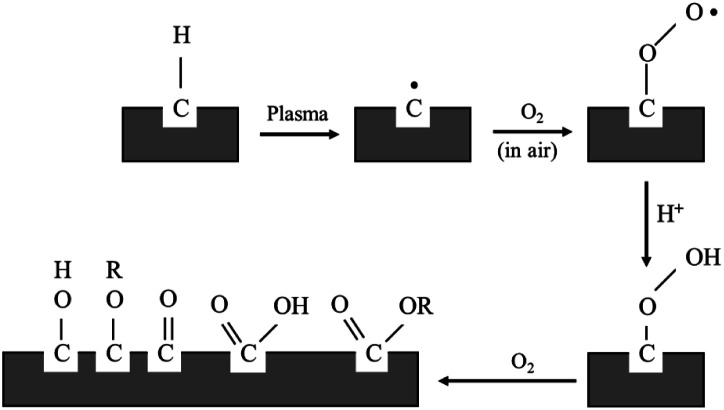
Mechanism of plasma surface treatment.^27^

For the purpose of this research work, the electroless plating technique was chosen for several reasons, for one it can be used to apply metal coating on an insulator substrate such as glass, plastic, and ceramic, while all of this can be achieved under low operating temperature (lower than boiling point of an electrolyte). In comparison to physical vapor deposition and chemical vapor deposition techniques,^26^ it is more suitable for an industrial process than the other methods because of its ease of application and reasonable costs. Copper cannot be directly coated on the surface of polypropylene composite because of its nonpolar surface that is inert to the reaction. Thus, surface treatment is necessary to modify a polar function group on the surface to create an active surface. Two effective treatments were rationally selected due to two contributing factors: first, it is a step that does not provide any complexity and second using non-decomposing the substrate. The process was starting with a plasma technique to vacuum the system. Then the argon gas was fed into the system, following that argon gas was activated to argon-ion using radiofrequency energy, respectively (Fig. 1). Next, argon ions were attracted to polypropylene composite surface resulting in a breakage of covalent bonds between C–C and C–H in polymer chains. The surface of polypropylene was then turned, attracting radicals on the polymer chains and reacting with oxygen or humidity. The functional groups containing an oxygen atom were formed on the surface of the polypropylene composite. The composite surface became more polar and acquired higher polarity property.

**Fig. 2:**
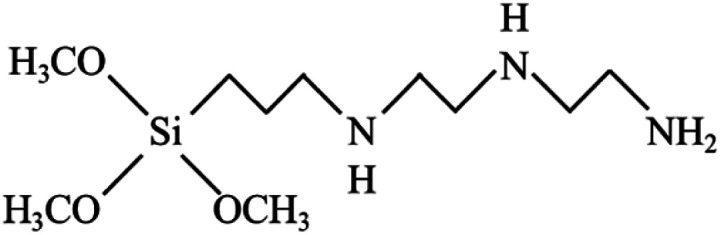
Molecular structure of *N*-3-(trimetylpropylsilyl diethylenetriamine: TMS).

To reach a goal of an effective adhesion between copper and the polypropylene composite, a silane coupling agent named *N*-3-tri(metylpropylsilyl)diethylenetriamine (TMS) was used in this experimental procedure. The molecular structure of TMS contains alkoxy groups such as the methoxyl group (–OCH_3_), ethoxyl group (–OCH_2_CH_3_) and the organo-functional group, as shown in Fig. 2.

**Fig. 3:**
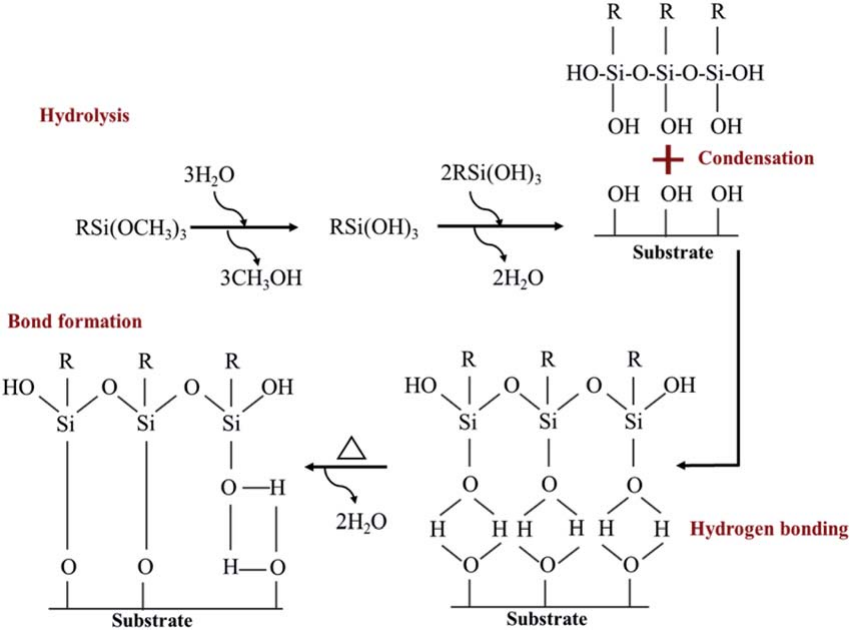
A self-assembled monolayer of silane molecule on hydroxyl functional surface.^28,29^

A hydrolysis, condensation, hydrogen bond and covalent bond formation were the chemical reactions created during the silanization process (Fig. 3).

Nitrogen in three amino groups of TMS shared electrons to with palladium(ii) ions (Pd^2+^), and a complex of Pd–N complex is formed. The catalyst (PdCl_2_) typically works in tandem with tin(ii) chloride (SnCl_2_) to increase the reaction rate, but excess tin(ii) ions (Sn^2+^) may form a gel film on catalyst surfaces.^30^ The gel formation is caused by catalyst agglomeration that contributes to poor catalyst distribution on the polymer surface. This gel film may shrink during a dehydration step contributing to the poor adhesion ability. Thus, only PdCl_2_ was used as a catalyst in this experimental work. The modified surface created from the silanization process, was coated with copper *via* electroless deposition, which is a redox reaction, as shown in eqn (3)–(5).^31^ Note that the source of copper was a copper sulfate solution (CuSO_4_·5H_2_O) stabilized by ligand (potassium-sodium tartrate). Copper layers on the composite surface were supposed to acquire 1.765 × 10^−8^ Ω to 7.536 × 10^−8^ Ω of electrical resistance.

3 Oxidation: HCHO + 3OH^−^ → HCOO^−^ + 2H_2_O + 2e^−^

4 Reduction: Cu_solution_^2+^ + 2e^−^ → Cu_lattice_

5 Redox: Cu^2+^ + 2HCHO + 4OH^−^ → Cu^0^ + 2HCOO^−^ + 2H_2_O + H_2_

The objective of this research is to reduce the surface resistance of a polypropylene composite bipolar plate using a copper electroless deposition technique. To accomplish this goal, the experimental activities can be separated into three main categories: composite preparation, bipolar plate fabrication, and characterizations which are laid out in the experimental methodologies sections of this research.

## 2. Experimental methodologies

### The fabrication of polypropylene composite bipolar plates

The BP fabrication can be separated into two main parts, which are polypropylene composite preparation and BP injection molding. The masterbatch of carbon black (CB: VULCAN® XC72 by Cabot Corporation)-filled PP (Equistar® PP35FU01) and synthetic graphite (from Asbury Graphite Mills Inc)-filled PP composites were prepared using a twin-screw extruder (*L*/*D* = 40, *D* = 27 mm; ZSE 27 Leistritz) with a side stuffer feeding. The process of mixing composite masterbatches with conductive fillers was carried out in a 270 mL mixing chamber of an internal mixer (Haake Fisons Rheocord 90). The specific conditions of composite mixing were as follows: 210 °C of melt temperature, 80 rpm screw speed, and 25 minutes of compounding time.

The 55 wt% of total filler load was formulated with different filler ratios, as illustrated in Table 1, since the composite containing higher filler loading than 55 wt% was not suitable to be injected in BP form. After the mixing process, the actual filler concentrations were determined using thermal gravimetric analysis (TGA). All formulated composites were injected in a bipolar plate and blank plate shapes using injection molding (Engel 85) with following operating conditions: 200 °C of melt temperature, 240 rpm screw speed, and 65 °C of mold temperature.

**Table:** Formulation of polypropylene composites

Composites	Filler loading (55 wt%)			
	PP (wt%)	Filler ratio		
		CB	CF	G
PPC 1	45.00	4.00	1.00	1.00
PPC 2	45.00	2.00	1.00	1.00
PPC 3	45.00	1.00	1.00	1.00

### Copper coating on polypropylene composite plates and BPs

**Fig. 4:**
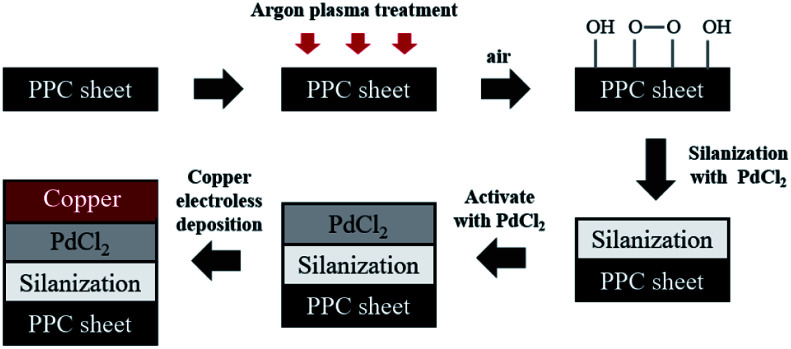
The schematic diagram of coating procedures.

Prior to the steps being taken, polypropylene composite (PPC) blank plates were cut in the dimension of 6.00 cm × 6.00 cm × 0.30 cm, next the surfaces of plates were cleaned with acetone using an ultrasonicating technique. The surfaces of cleaned PPC plates were also treated with plasma treatment in Harrick PDC-32G operated using 18 watts of power setting, utilizing argon gas. After plasma treatment, PPC plates were left in the normal atmosphere approximately 10 minutes for creating polar functional groups on PPC surfaces. The modified PPC plates were immersed into 4.00 wt/v% of TMS solution (analytical grade from Sigma-Aldrich) for 30 minutes to generate the silanization reaction. Nitrogen purge drying for 10 minutes is advisable to achieve surface dehydration for forming Si–O–C bond, to facilitate solvent removal, and to achieve chemical stability.^32,33^ Then silanized PPC plates were cleaned using 1,4-dioxane (analytical grade from Fisher Scientific) and deionized water. Next, the silanized PPC plates were coated with palladium(ii) chloride (PdCl_2_: analytical grade from Sigma-Aldrich) catalyst to achieve surface activation. The plates were dipped in 0.03 wt/v% PdCl_2_ solution prepared in 1.00 wt/v% of hydrochloric acid solution for 10 minutes. Then activated plates were cleaned with deionized water and purged by nitrogen gas. The copper solution used for the coating step was prepared by blending 1.50 wt/v% of copper sulfate pentahydrate (CuSO_4_·5H_2_O), 3.50 wt/v% of potassium sodium tartrate (KNa-tartrate), and 2.00 wt/v% of sodium hydroxide (NaOH). Formaldehyde (CH_2_O) from Sigma-Aldrich was added to the copper solution to create a redox reaction, and then PPC plates were immersed in the copper solution for 5 minutes. Finally, the copper-coated PPC plates were cleaned again by deionized water and purged with nitrogen gas. In the case of copper coating on polypropylene composite BPs, all procedures were composed using the same method as the copper coating on PPC plates. The schematic diagram of coating procedures can be seen in Fig. 4.

### Characterizations

Modified PPC plates were chemically characterized using three techniques; by observation of surface for hydrophilic property using contact angle goniometer, studying chemical characteristics of the surface using Fourier-transform infrared spectroscopy (FTIR: Perkin-Elmer, Spectrum One) and X-ray photoelectron spectroscopy (XPS: Kratos Axis Ultra DLD spectrometer), to confirm that the copper coating process was successful. An adhesion test using a paint adhesion test kit (Paul N Gardner, PA2000) based on ASTM D 3359 and the surface morphology observation *via* scanning electron microscope (SEM: JSM 5410 LV, Oxford EDS) were carried out to evaluate the adhesive performance between two interfaces. In addition to that, cathodic delamination (CD) was evaluated through electrochemical impedance spectroscopy (EIS) using potentiostat galvanostat (Autolab) to study the effects of a DMFC in an operating environment on adhesion layer between a copper and PPC surface. The surface conductivity of coated plates was determined by a four-point probe (SP4) and source meter (2400C) to make sure that coating copper can deduct the electrical surface resistance on the composite surfaces. A single cell of DMFC was constructed from a commercial membrane electrode assembly (MEA: Nafion 117) with 4 mg cm^−2^ of Pt/Ru catalyst at the anode side and 4 mg cm^−2^ of Pt black catalyst at the cathode side, Teflon™ gasket, and SGL gas diffusion layer (woven carbon fiber cloth). The thickness of the bipolar plates was 3 mm, with an active cell area of 16 cm^2^. Methanol solution (1.00 mol L^−1^) and air (1.0 L min^−1^) were fed to the anode and cathode, respectively. The performance of the single-cell was evaluated by measuring the *I*–*V* characteristics using an electronic load.

## 3. Results and discussion

### Effect of plasma treatment time on the change of the contact angle

Polypropylene composites (PPCs) containing carbonaceous fillers have hydrophobic characteristics because a basic repeating unit of polypropylene is the only hydrocarbon and while fillers only have carbon content. A hydrophobic PPC plate has poor wettability and adhesion. Particularly if it is coated by a polar material such as copper, the problems like poor adhesion and easy detachment will occur. This is due to the variation of surface free energy in the interaction of two materials, which is quite large. In the first stage of this research work, a series of experiments were carried out by a process known as atmospheric pressure plasma. In order to coat the surface of PPC plates with copper, a reaction in the water solvent must take place. Therefore, the surface of the PPC plates must be polarized. First, the electrical energy is applied from a plasma chamber to dissociate the inert argon gas into electrons, free radicals, ions, photons, and metastable species. These free radicals and electrons generated in the plasma chamber collide with the PPC surface, which results in covalent bonds being broken. Free radicals produced on the surfaces of PPC plates associate with oxygens and moistures in the atmosphere to deliver thermodynamically preferred functional groups on the PPC surfaces (Fig. 1).

**Fig. 5:**
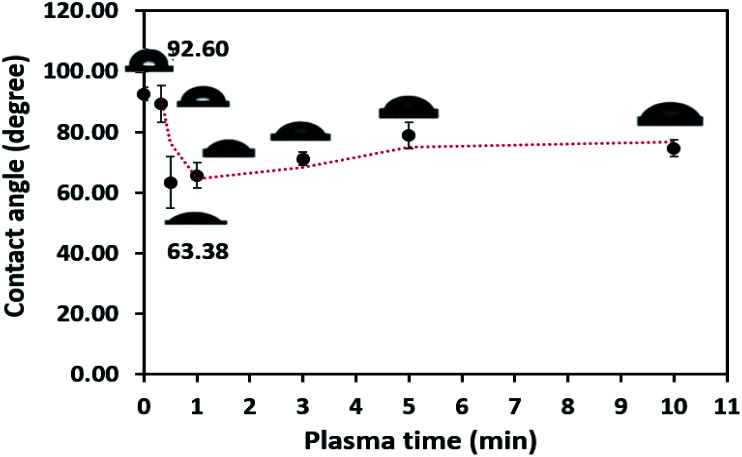
Contact angle variation as a function of plasma treatment time.

The obtained water contact angle results depending upon plasma the time of treatment (from 20 s to 10 min) are illustrated in Fig. 5. The results indicate that at 0–30 seconds of treatment time, the contact angle decreased from 92.60° ± 2.26° to 63.38° ± 8.54°. The decrease in contact angle confirms the contact angle change (92.60° to 60.00°) stated by Morent.^34^ It can be ascribed that the plasma activation method can create polar groups on the PPC surface. Consequently, plasma treatment time for 30 seconds is an appropriate time for the plasma process.

**Fig. 6:**
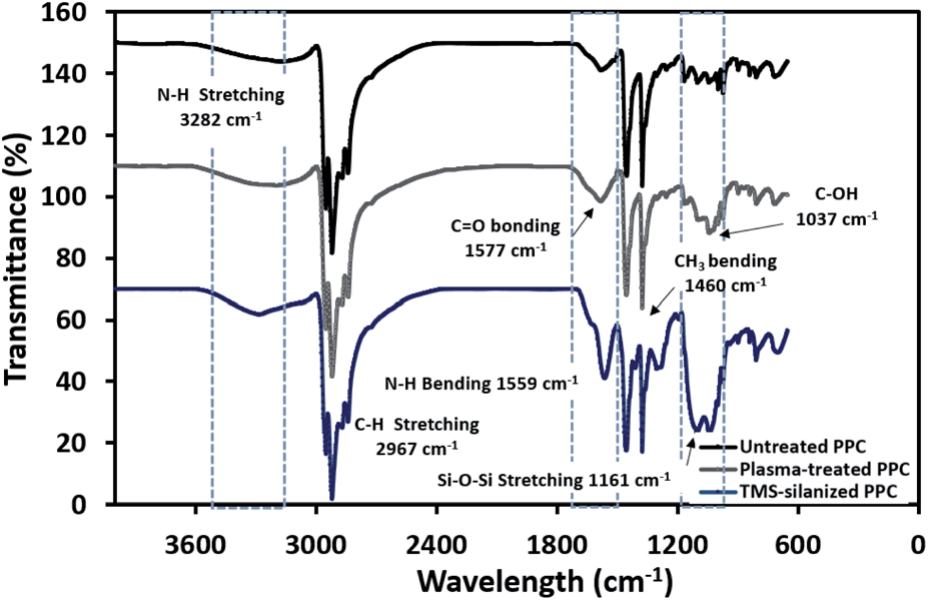
FTIR spectra of untreated PPC and treated PPC in each step.

The physicochemical changes after surface modification *via* the plasma process were elucidated using ATR-FTIR to support the results from contact angle observation (Fig. 6). The spectra of untreated PPC and plasma-treated PPC indicate that both of them possess peaks of –CH_3_ at 2967 and 2972 cm^−1^, –CH_2_– at 2839 and 2920 cm^−1^, and CH_3_ bending at 1460 cm^−1^. Primary alcohol stretching (C–OH) was located at 1037 cm^−1^ after plasma treatment, while this peak was not observed in untreated PPC. Furthermore, the spectrum evidently displayed the peak of the carbonyl group of carboxylic acids/derivates (C=O vibration) located at 1577 cm^−1^. Both results from ATR-FTIR and contact angle measurements confirmed that the PPC surface could be modified by plasma treatment.

**Fig. 7:**
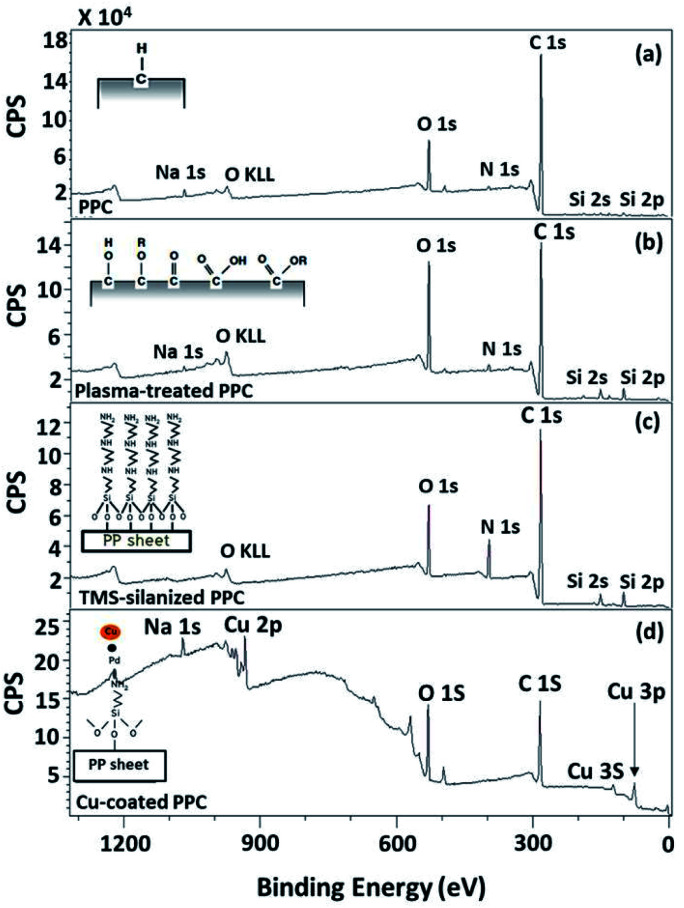
Wide scan XPS spectra of (a) untreated PPC and the treated PPC surfaces by (b) plasma activation, (c) TMS silanization, and (d) Cu-coating.

To evaluate existing functionalities on PPC surfaces before and after the plasma treatment, wide scan and high-resolution XPS spectra were recorded, as shown in Fig. 7.

The elemental composition of the PPC surfaces was calculated from the XPS spectra. The spectra showed that the PPC surface consisted of C 1s which is a polypropylene component, but H peak could not be found due to high binding energy. The signals of N 1s, Na 1s, Si 2s, and Si 2p would be the components of additive. Plasma influenced on O 1s increasing peak intensity from 11.76% to 19.98%, while the quantity of C was reduced from 84.84% to 72.11% (Table 2). This is because free radicals formed during the plasma process existed on the PPC surface and reacted with oxygen and moisture to produce functional groups containing O element; therefore, the peaks of these groups may overlay the C peak.

**Table:** Atomic concentration percentage derived from wide scan XPS spectra

Processes	Atomic concentration (%)					
	C	O	N	Si	Na	Cu
Untreated PPC	84.84	11.76	0.91	1.93	0.56	—
Plasma-treated PPC	72.11	19.98	1.65	5.97	0.29	—
TMS-silanized PPC	72.86	11.67	9.15	6.31	—	—
Cu-coated PPC	62.49	27.63	—	—	3.15	6.72

**Fig. 8:**
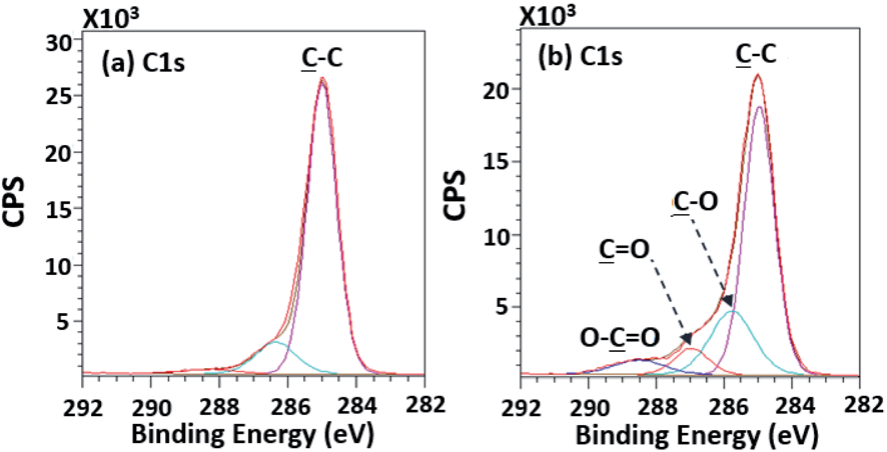
High-resolution XPS spectra of (a) the untreated PPC and (b) the treated PPC surfaces by plasma activation.

According to high-resolution spectra, the peaks of O=C, O–C, C–C, and O–C=O were found to confirm that the treatment was a success (Fig. 8). The intensities of O=C, O–C, and O–C=O peaks of plasma-treated PPC obviously increase (Fig. 7b and Table 3), compared to untreated PP-CF (Fig. 7a and Table 3). On the contrary, the C–C content, decreased due to polar functional group interacting on the surface of PPC occuring to the plasma treating.^23^ Carbon free radicals created after the plasma treatment process reacted with oxygen and humidity in an atmosphere, functional groups consisting of oxygen atoms were subsequently formed. The carbon atomic concentration ratios of C̲–O/C̲–C, C̲=O/C̲–C, and O–C̲=O/C̲–C on the surface of plasma-treated PPC significantly increased from that ratio in untreated PPC.

The C 1s intensities found from untreated PPC and treated PPC surfaces

**Table:** 

Samples	Atomic concentration (%)			
	C̲–C	C̲–O	C̲=O	O–C̲=O
Untreated PPC	75.01	11.28	—	1.97
Plasma-treated PPC	51.71	17.57	5.63	4.58
TMS-silanized PPC	44.41	23.98	5.21	—
Cu-coated PPC	38.74	20.01	2.28	7.02

**Table:** 

Samples	Ratio (%)		
	C̲–O/C̲–C	C̲=O/C̲–C	O–C̲=O/C̲–C
Untreated PPC	0.15	—	0.03
Plasma-treated PPC	0.34	0.11	0.09
TMS-silanized PPC	0.54	0.12	—
Cu-coated PPC	0.52	0.06	0.18

### Physicochemical changes after silanization

**Fig. 9:**
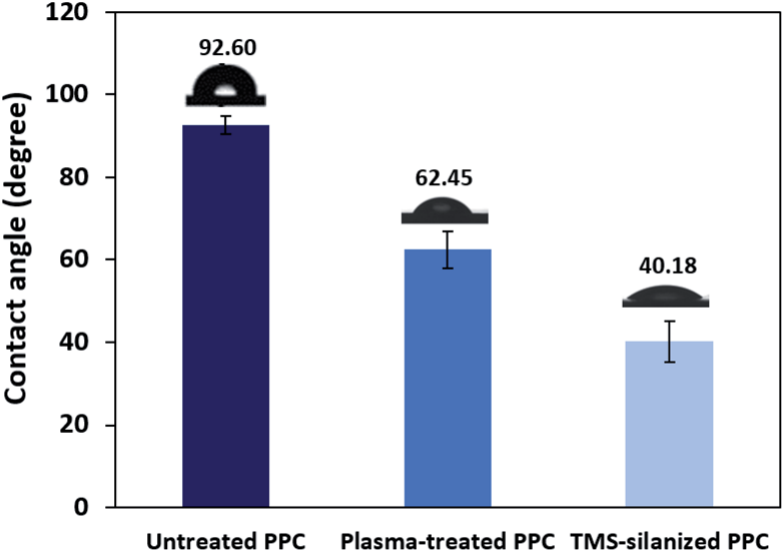
The contact angle of untreated PPC, plasma-treated PPC and TMS-silanized PPC surfaces.

TMS was used to increase the potential of the adhesion between copper and PPC interfaces. TMS created a covalent bond and charged functional groups on the plasma-treated PPC surface, as in Fig. 3. The contact angles of PPC surfaces after the plasma treatment and TMS- silanization were observed to make sure that the PPC plate occupied a hydrophilic surface. The hydrophilicity of PPC surface is necessary for the copper coating step, since the reaction is driven in an aqueous copper solution. The contact angle of plasma-treated PPC decreased from 62.45° to 40.18° (Fig. 9), and the results were similar to that of Phasuksom's,^23^ which indicated contact angle change (40.00–60.00°) after coating fluorinate ethylene propylene with TMS. Note that the contact angle of copper-coated surface was approximately 86°.^35^

The spectra of the silanized PPC surface exhibited the peaks of N–H stretching at 3282 cm^−1^, N–H bending at 1559 cm^−1^, and Si–O–Si at 1161 cm^−1^ (Fig. 6). These peaks represent functional groups in TMS. Addressing the peak area of Si–O–Si, this area was enlarged after a silanization reaction due to the reaction mechanism that took place, which is explained in Fig. 3. Wide scan XPS spectra confirmed that the silanization was completed (Fig. 7c). When comparing the wide scan XPS spectra of PPC plates before and after the silanization with TMS, the N 1s content from XPS dramatically increased from 1.65% to 9.15% (Table 2). It means the nitrogen content increased by the amino groups in TMS silane. On the other hand, the oxygen content decreased from 19.98% to 11.67%, because the oxygen element was obscured by the coated TMS silane compound, resulting in the reduction of oxygen content. Si content, a component of TMS silane, also appeared in the spectra as illustrated in Fig. 7c. Even the Si peaks in XPS spectra were small, the calculation of elemental composition (Table 2) provided the evidence that Si content on the TMS-silnized PPC surface was increased around 4.38% compared to untreated PPC. Basically, silicon could be everywhere as a contaminate, therefore; N content was needed to be considered in parallel. From the Table 2, nitrogen content intensely increased after silanization. This can indicate the successful silanization.

**Fig. 10:**
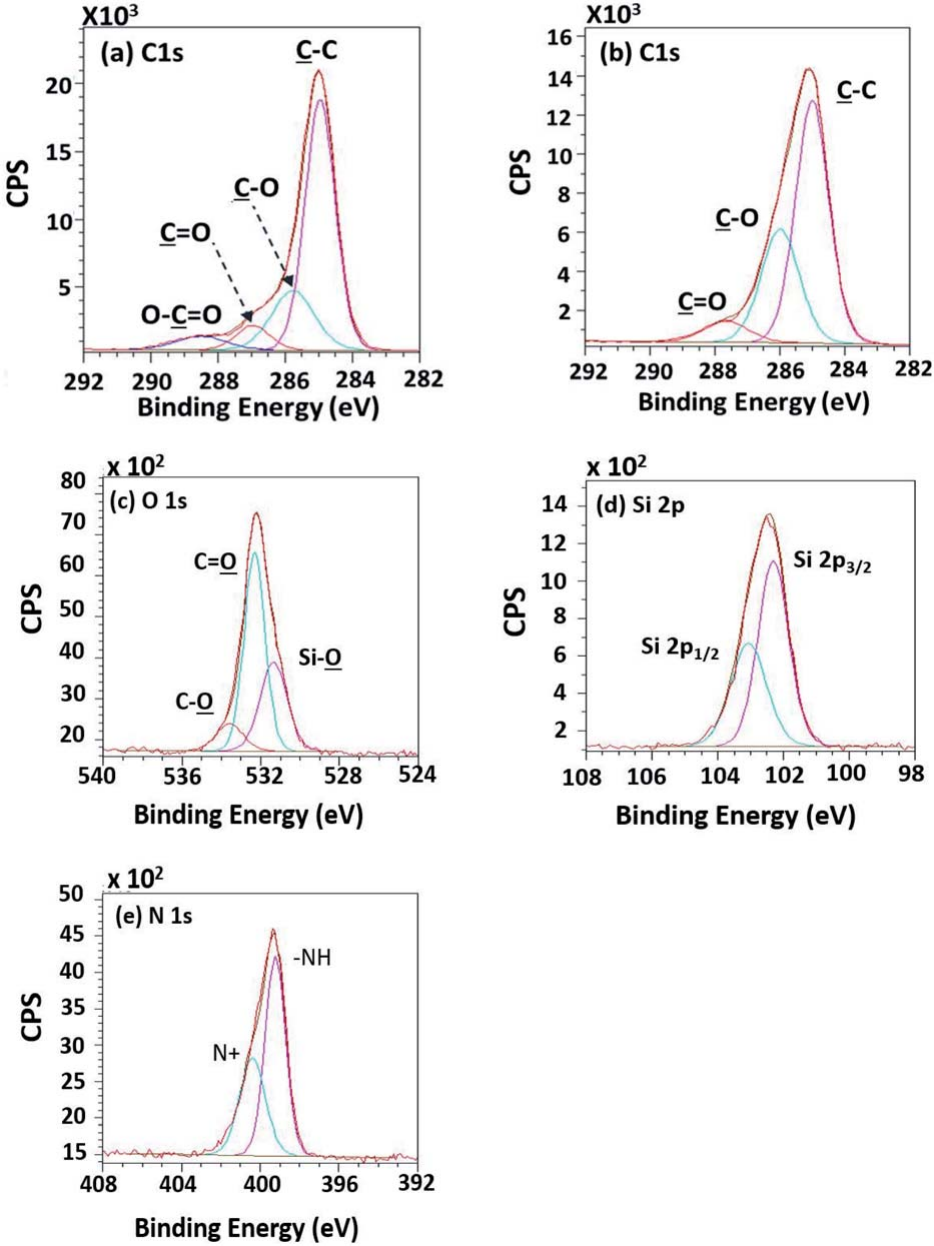
High-resolution XPS spectra of (a) the unsilanized PPC and (b–e) the silanized PPC surfaces by TMS.

High-resolution XPS spectra in Fig. 10 show interesting functional groups and species comprising of Si 2p_1/2_, Si 2p_3/2_ (TMS component), silanol group (Si–O), –NH–, and N^+^. The emergence of the TMS silanization caused those species.

Pd in high resolution mode was not diagnosed in this work, but results from our previous work presented Pd 3d peaks indicating Pd–N complex signal at lower binding energy tail.^36^ Both SnCl_2_ and PdCl_2_ have commonly been used in the activation process. The palladium ions (Pd^2+^) are adsorbed and reduced to Pd^0^ by SnCl_2_.^37^ SnCl_2_ in activation process typically acts as reducing agent for Pd^2+^ as follows:

6 Sn^2+^ + Pd^2+^ → Pd^0^ + Sn^4+^

Recently, Sn-free activation processes have been developed following economic and environmental considerations. It has been reported that palladium may be adsorbed directly on the nitrogen functional groups of the polymer surfaces, allowing the electroless deposition of metals without the use of SnCl_2_.^38,39^ In the mentioned previous work,^36^ the Pd 3d high-resolution XPS spectrum of the silanized surface after PdCl_2_ activation showed the evidence of Pd–N complex at higher biding energy tail (Pd 3d_5/2_ = 336.2 eV and Pd 3d_3/2_ = 341.4 eV). The nitrogen atoms of the silanized surface can attract the palladium ions from the PdCl_2_ solution by sharing their lone pair electrons, leading to Pd–N complex. In the case of this work, Sn-free process was applied, therefore Pd^2+^ was not reduced to Pd^0^, but form Pd–N complex instead. However, the coordinated Pd–N complex can be reduced to Pd metal in the alkaline solution of the plating bath.^39^ The reduced Pd is utilized subsequently to catalyze the electroless plating of copper.

### Copper synthesis on composite polypropylene plates

Before copper synthesis on PPC plates, the plates were coated with a PdCl_2_ catalyst to decrease the activation energy of copper coating reaction. The copper coating reaction mechanism was mentioned in Fig. 4. The most important parameter for an electroless deposition was operating temperatures which should be controlled in the range of 30.0–60.0 °C. In this work 28.0 °C, 40.0 °C, and 70.0 °C of operating temperatures were varied to investigate an operating temperature effect on a copper-coated surface characteristic. The coating process could not be completed if the process of the electroless deposition was done under temperature lower than 30.0 °C (at 28.0 °C in this scenario). When the temperature was higher than 60.0 °C (at 70.0 °C for this case), the colour of the copper solution changed from blue to pink, causing the PPC surface to become dark brown (Table 4). This is caused by decomposed^40^ formaldehyde in the copper solution.

**Table:** The colour changes copper coated-surfaces when copper coating processes were operated at different temperatures

Temperature for coating (°C)	Cu-coated PPC
(a) 28.0	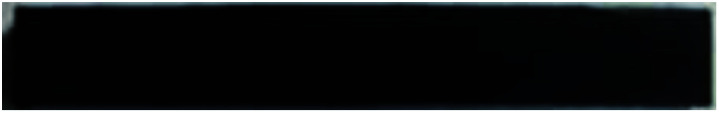
(b) 40.0	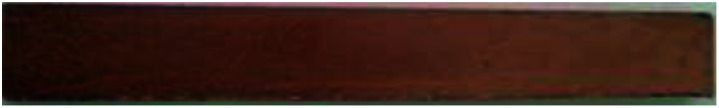
(c) 70.0	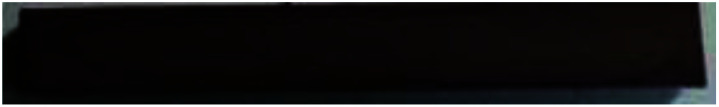

Wide scan XPS spectra, a copper-coated PPC plate showed copper occurrence from the peaks of Cu 3s, Cu 2p and Cu 3p (Fig. 7d), which clearly showed that copper certainly adhered to the PPC surface. An existing sodium element which is related to K-Na tartate content, a ligand in the copper solution. As for the oxygen content, the quantity of oxygen increased from 11.76% to 27.63% (Table 2). It is worth noting, however, that copper was not the only element that deposited on the PPC surface, but copper oxide also coated the surface of PPC as well.

**Fig. 11:**
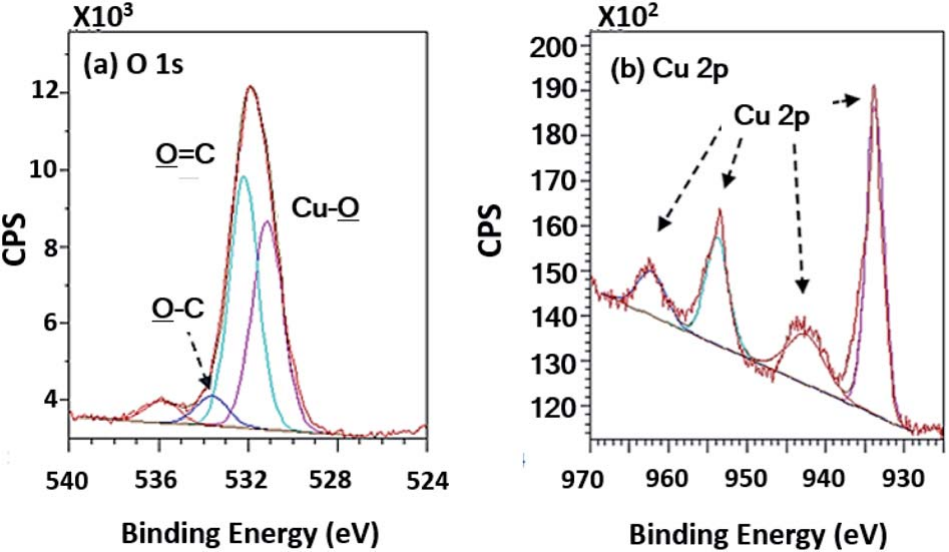
High-resolution XPS spectra of the copper coated PPC surfaces.

In terms of high-resolution XPS (Fig. 11), the spectrum indicated a double peak of Cu 2p, and Cu–O̲ appearing in the spectra of O 1s at 531.15 eV of binding energy.

The reaction rate typically depends on copper ion concentration, as shown in eqn (7). Hence, the concentration of the copper ion influences kinetic and mass transfers.^31,40^

**Fig. 12:**
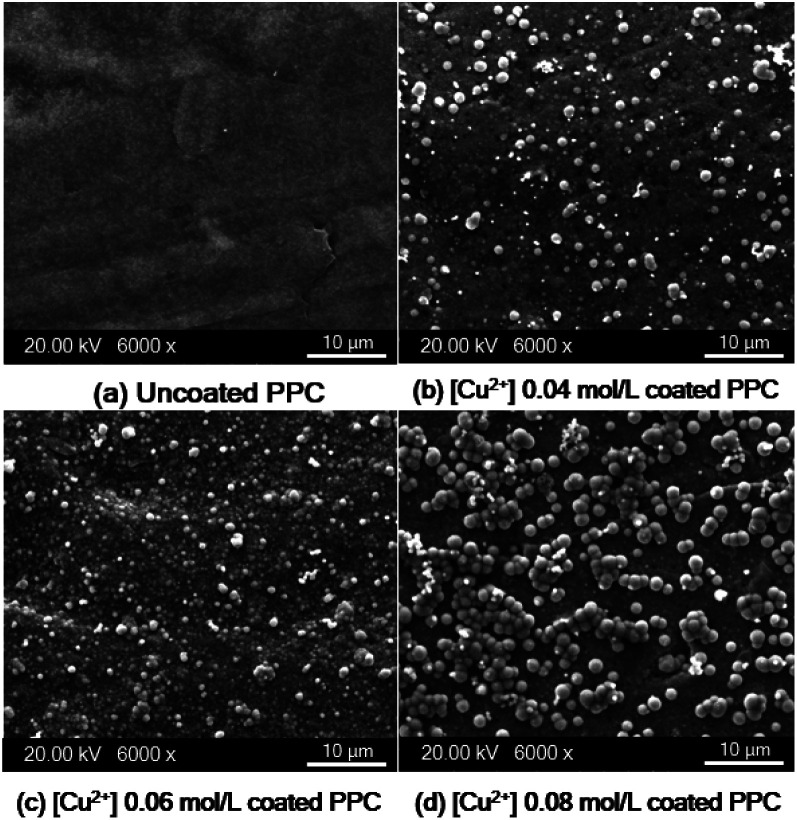
SEM images of copper-coated on PPC surface with different Cu^2+^ concentration (a) uncoated surface (b) 0.04 mol L^−1^ (c) 0.06 mol L^−1^ (d) 0.08 mol L^−1^.

**Fig. 13:**
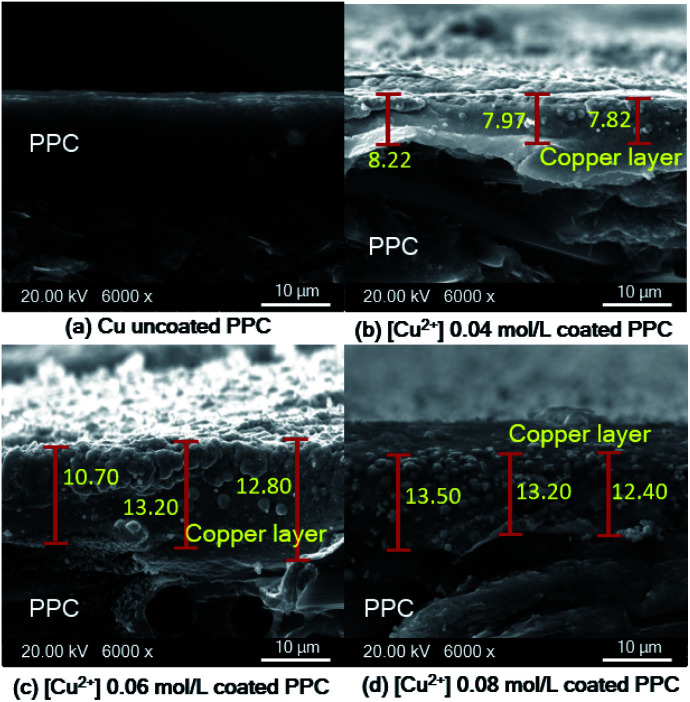
SEM cross-section images of copper-coated PPC plates with different Cu^2+^ concentration (a) uncoated surface (b) 0.04 mol L^−1^ (c) 0.06 mol L^−1^ (d) 0.08 mol L^−1^.

7 *r* = *k* [Cu^2+^]^*a*^ [OH^−^]^*h*^ [HCHO]^*c*^ [LIGAND]^*j*^

in which *r* is reaction rate, *k* is reaction rate constant, [Cu^2+^] is concentration of Cu^2+^, [OH^−^] is concentration of OH^−^, [HCHO] is concentration of HCHO, and [LIGAND] is concentration of ligand. Different concentrations (0.04, 0.06 and 0.08 mol L^−1^) of copper solution were prepared to study the effects of Cu^2+^ concentration on copper coating ability. When Cu^2+^ concentration was lower than 0.01 mol L^−1^, the Cu^2+^ concentration in the solution was slightly different from the Cu^2+^ concentration on the PPC surface, which contributed to a low mass transfer process.^40^ Using the Cu^2+^ concentration in the range of 0.01 mol L^−1^ < Cu^2+^ < 0.20 mol L^−1^ increased Cu^2+^ diffusion rate of the coating process. SEM images show deposited copper particles on the PPC surface (Fig. 12a–d). Copper particles provided a better coating degree with the increase in Cu^2+^ concentration from 0.04 mol L^−1^ to 0.06 mol L^−1^. Even the efficiency of the copper coating was at its highest with 0.08 mol L^−1^ of Cu^2+^ concentration. The incomplete coating appears to have been caused by the copper particles agglomeration (Fig. 12d). The thickness of the copper layer was differentiated by variation of Cu^2+^ concentration, as illustrated in Fig. 13.

As shown in SEM images, the thickness of the top layer (10–15 μm) probably include both copper layer and TMS-silanized layer underneath. Even electroless plating is regularly used to prepare metal seed layer of approximately 1 to 3 μm, some publications reported that metallic layer generated from electroless plating process typically was varied in the range of 2-50 μm.^41^ Y. H. Lee, *et. al.*^8^ coated cooper onto polycarbonate substrate *via* the electroless deposition technique, and the results indicated that average thickness of copper layer was around 10 μm. The quantity of active areas and reaction time for the electroless deposition are important factors influencing the thickness and peeling.^41,42^ The thickness of a metallic layer will increase with longer reaction time.^42,43^ According to surface treatment step through silanization, literature stated that the thickness of silane layer was in the range of 0.5 to 2.5 nm.^44^ To make more clear comprehension, the thickness of coated layer should be investigated in depth using white-light interferometer or scanning electron microscope for further work.^45^

In terms of a bipolar plate application, suitable thickness is relevant to the depth of reactant flow channels located on a BP surface and copper layer peeling off. The depth of flow channels for a fuel cell application was typically imposed in the range of 0.50–0.53 mm,^46^ but some research work designed the channel depth approximately 0.760 mm and 1.00 mm.^47,48^ The channel depth influences on fuel cell performance. Lower channel depth causes to faster reactant flow leading to higher reaction rate according to the Sherwood number.^49^ Furthermore, the lower channel depth reduces the ohmic loss of BP as a following equation.8
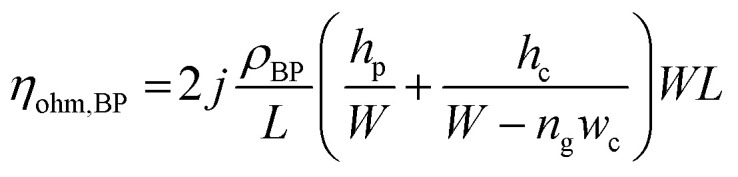


In accordance with the above equation, *ρ*_BP_ is resistivity of BP, *L* and *W* are the length and width of the gas flow channel plate, and *n*_g_ is the number of gas flow channel. The *h*_p_ and *h*_c_ are the thickness of the plate and height of the gas flow channels, respectively.^50^ The reactant flow channels designed for this work was 0.75 mm, thus the depth copper-coated channels were around 0.735 mm which was normal for the fuel cell application. It implies that the channel can be coated with thicker layers as a multilayer coating process, however; coated-layer thickness affects the adhesion ability. Thicker copper-coated layer may be easily peeled in comparison to a thinner layer,^51^ since agglomeration of copper particles piles up to form the copper layer. In brief, 0.06 mol L^−1^ of Cu^2+^ concentration was used to coat PPC plates under 40.0 °C of operating temperature.

### Possibility of using the copper coated composites as bipolar plates

**Fig. 14:**
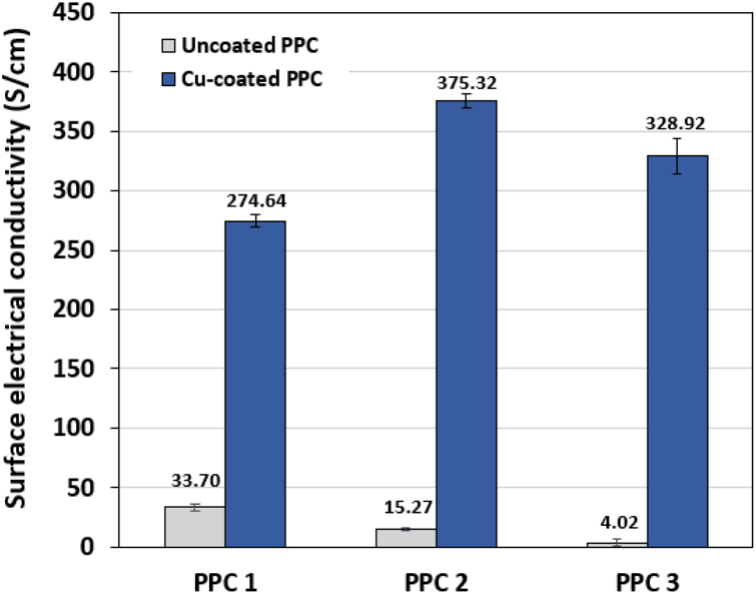
The surface electrical conductivity uncoated and copper-coated composite plates.

**Fig. 15:**
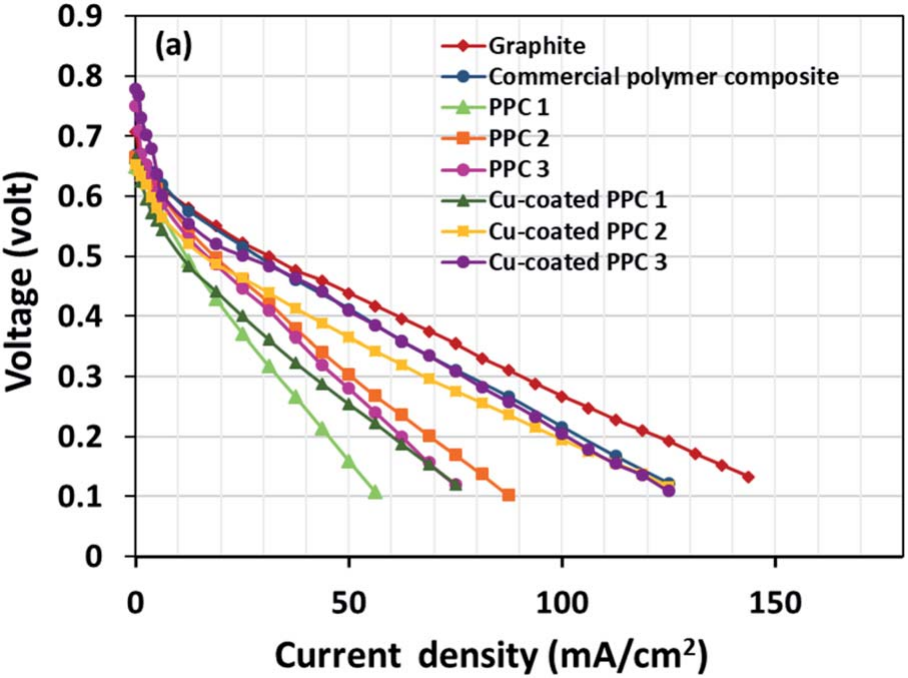
The polarization curves of DMFCs assembled with a different bipolar plates.

Decreasing the electrical resistance of the surface or increasing its electrical conductivity is a necessary strategy when it comes to developing BPs since one of the key properties of BPs is “electrical conductivity. The electrical conductivity of the surface of three composites (PPC 1, PPC 2, and PPC 3) were 33.701, 15.274 and 4.023 S cm^−1^, respectively (Fig. 14). After coating copper on the surfaces of these composites, the conductivity increased to 274.64 S cm^−1^, 375.32 S cm^−1^, 328.92 S cm^−1^. All values achieved and met the requirement of the USA-Department of Energy (100 S cm^−1^) for a BP application. The results indicated that the ratio of carbon fillers had a direct effect on coating performance. The different filler-shape combination may provide various surface porosity and roughness composite surfaces. Surface structure of BPs was suggested to be monitored in depth using atomic force microscopy, since research publications reported substrate surface roughness and porosity on coating surface ability and electrical resistivity reduction.^2,52^ Volume electrical conductivity or through-plane conductivity was also measured. PPC 1, PPC 2, and PPC 3 provided 1.20, 0.69, and 0.47 S cm^−1^ of the volume electrical conductivity. Uninsulated behaviour of composite BPs in a through-plane direction was underpinned by these measured data. *In situ* DMFC performance was validated to investigate the possibility of using invented BPs in an actual DMFC. The in-house DMFC was operated under an operating temperature of 80.0 °C, then cell voltage and current density were used to plot the polarization curve (Fig. 15).

**Fig. 16:**
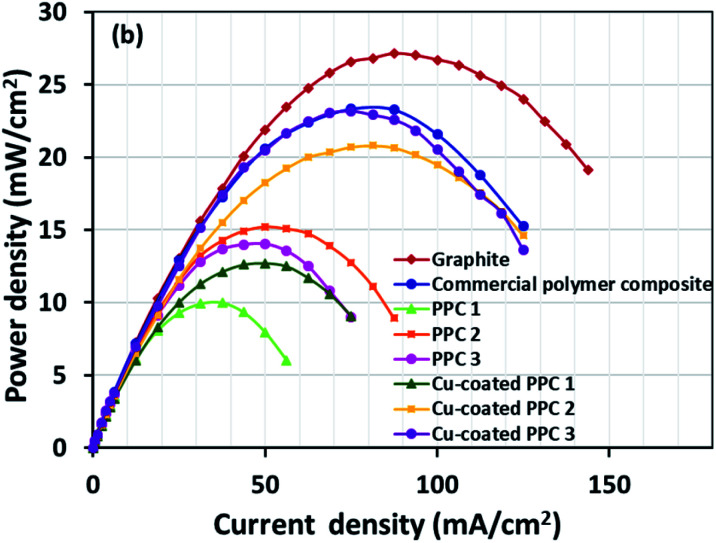
Power density curves of DMFCs assembled with a different bipolar plates.

The cell performance of DMFC assembled with copper-coated BPs was superior to the performance of that of a DMFC using uncoated BPs. It can be deduced that the copper layer can reduce the ohmic loss, since the surface resistance was decreased. Surprisingly, copper-coated PPC 3 provided the highest performance with 23.18 mW cm^−2^ (Fig. 16) of power density, though the copper-coated PPC 2 had the highest electrical conductivity. It seems that it related to the peeling of the copper layer in the DMFC environment. When it comes to cell performance, the copper-coated PPC 3 is a promising proposition to be used as a commercial composite BP since the performance was equal to that of a commercial composite BP made from an epoxy composite (Fig. 15, 16 and Table 5). Regardless of that, its performance is still inferior to a commercial graphite BP. The efficiency of DMFC, consisting of invented BP, can be calculated using eqn (9). The efficiency at an open-circuit voltage (OCV) of DMFC assembled using copper-coated PPC 3 was 60.91%, while the cell with graphite BPs provided 59.26% of the efficiency.9
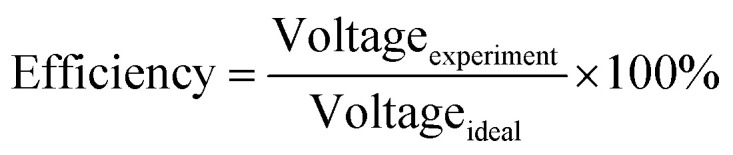


**Table:** Important data determined from DMFC performance test

Types of bipolar plates	Open circuit voltage (V)	Limiting current (mA cm^−2^)	Max. power density (mW cm^−2^)	Efficiency (%)
Graphite	0.717	143.75	27.13	59.26
Commercial polymer composite	0.770	125.00	23.33	63.64
PPC 1	0.728	56.25	9.975	60.17
PPC 2	0.752	87.50	15.20	62.15
PPC 3	0.744	75.00	14.05	61.49
Cu-coated PPC 1	0.700	75.00	12.70	57.85
Cu-coated PPC 2	0.694	125.00	20.80	57.36
Cu-coated PPC 3	0.737	125.00	23.18	60.91

Copper layer delamination and surface corrosion are significant issues when it comes to the reliability of DMFC;^53^ thus, adhesion and corrosion tests were performed in this research. The results from the adhesion test were presented in Table 6 which displays surfaces of tapes and coated plates after the tests. If the tape surfaces own copper trace, that means a copper layer was removed. Peeling levels were determined by traces per area. Results showed that the percentage of copper layer removal was in the range of 5–15% (3B standard). If PPC plates were not primarily treated by plasma, the percentage of copper removal increased to around 65% (0B standard). The plasma treatment dominated a strong effect on adhesion ability.

**Table:** The adhesion test results according to ASTM D3359

Samples	Before adhesion test	After adhesion test		Results
		Tape surfaces	Sample surfaces	
PP	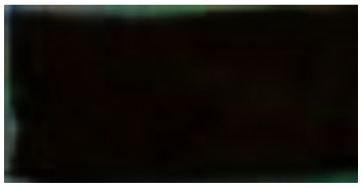	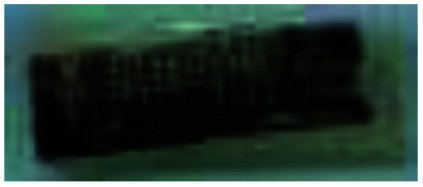	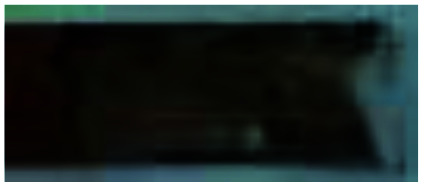	0B
Untreated PPC	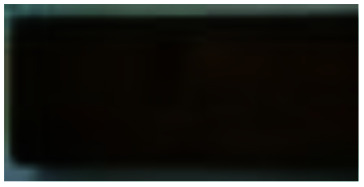	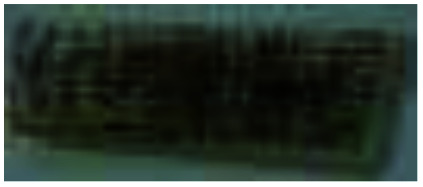	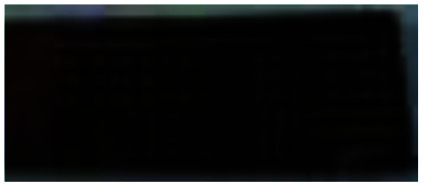	0B
Plasma-treated PPC	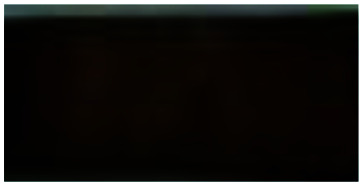	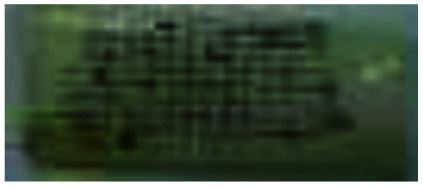	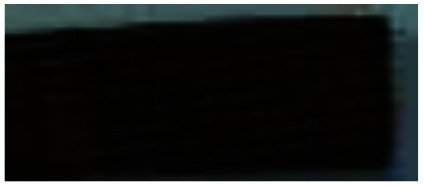	3B

Copper layer delamination or corrosion will cause cell voltage degradation; therefore, the corrosion test in the circumstance of DMFC operation was of utmost importance. After the fuel cell performance test was finished, incomplete copper covering could be observed, which was caused by copper layer delamination (Table 7).

**Table:** Surface feature of copper-coated BPs before and after DMFC operation

Samples	Before cell operation		After cell operation	
	Anode	Cathode	Anode	Cathode
PPC 1	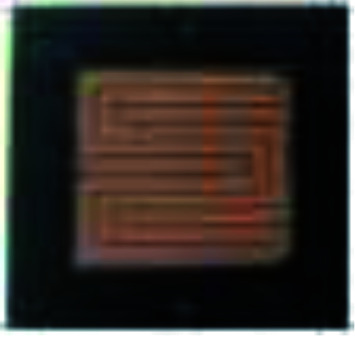	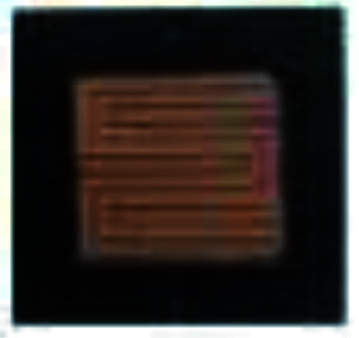	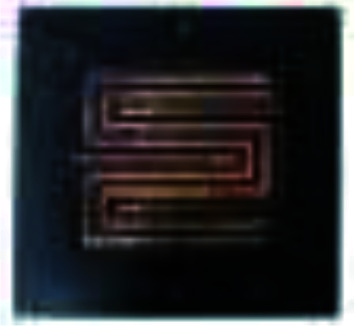	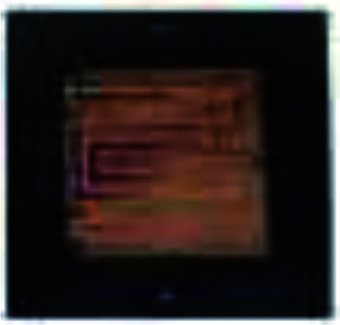
PPC 2	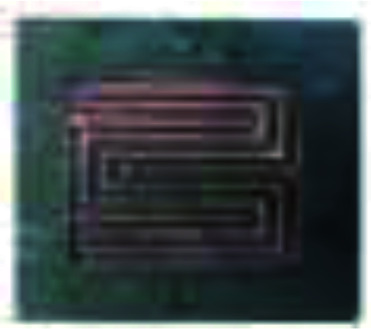	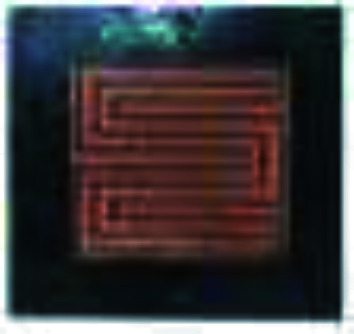	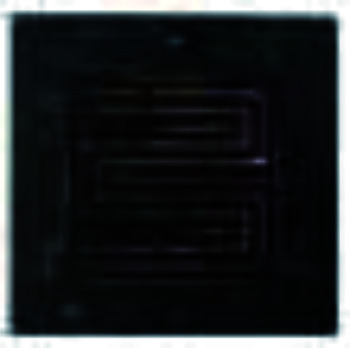	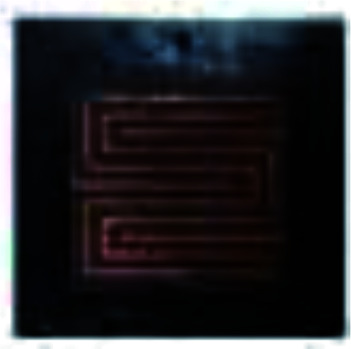
PPC 3	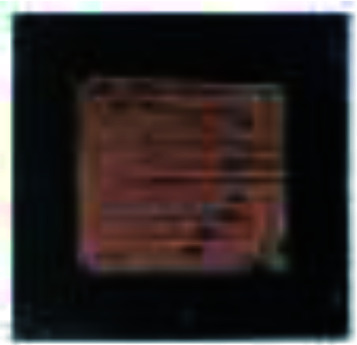	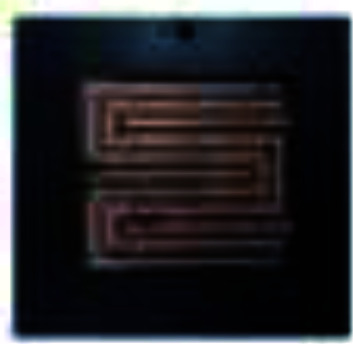	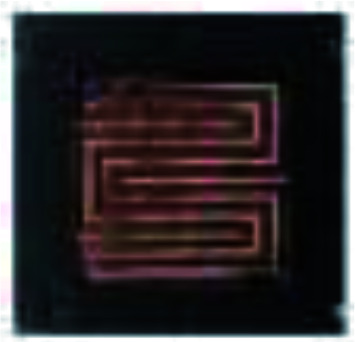	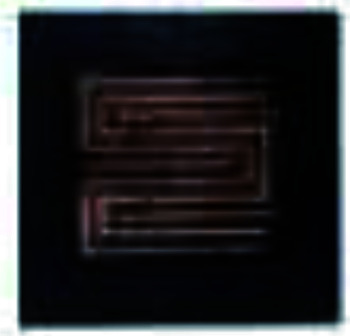

Moreover, the colour of the copper layer changed to green (copper(ii) oxide), which was caused by a copper reaction with methanol on the anode side (eqn (10)). At the cathode side, the air zero which is oxidant reaction with copper layer produced copper(i) oxide which is pink (eqn (11)). The picture in Table 7 displays colour changes of the copper layer after the DMFC operation.

10 Cu + CH_3_OH → CuO + CH_4_

11 4Cu + O_2_ → 2Cu_2_O

**Fig. 17:**
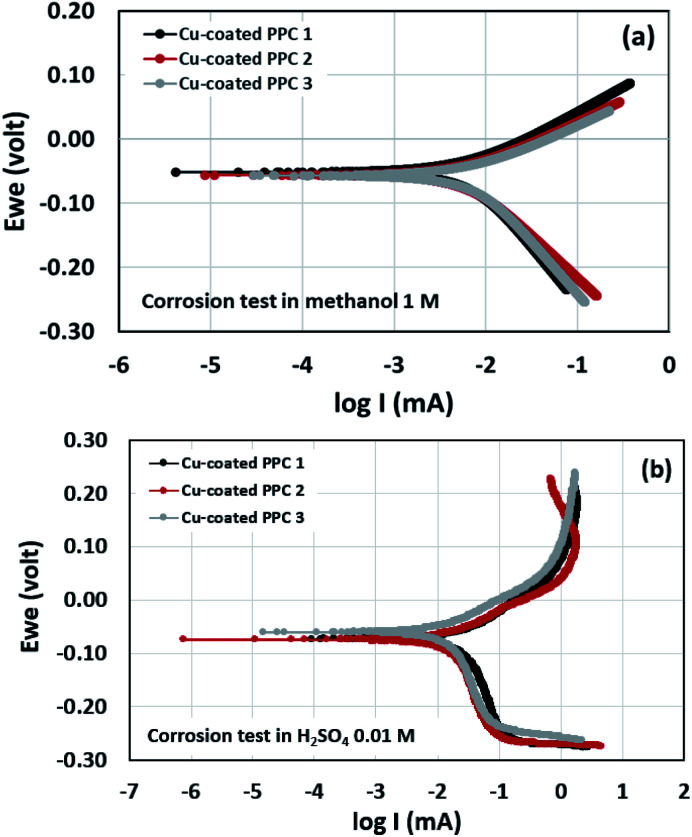
Polarization curve of copper-coated BPs in (a) 0.01 M H_2_SO_4_ and (b) 1.00 M CH_3_OH.

Potentiodynamic polarization measurements were performed in determination of surface corrosion in harsh environment. Copper-coated PPCs were submerged in 1 M methanol and 0.01 M H_2_SO_4_ for corrosion tests (Fig. 17). The 1 M methanol was a reactant used for operating DMFC, while 0.01 M H_2_SO_4_ gave pH equal to 2 which is a cell operating atmosphere. The corrosion current values can be determined from cathodic tangent slope and corrosion potential.^54^ Corrosion rates of copper-coated PPCs in a year were calculated as illustrated in Table 8 and 9. The testing results in 1 M methanol solution demonstrated that copper-coated PPC 3 had the lowest *E*_corr_ and the highest *I*_corr_ corrosion rate as shown in Table 8. Copper coated PPC 3 was easily corroded; the copper layer was in a situation where oxidation reaction can occur.

**Table:** Corrosion factors determined from the test in 1 M CH_3_OH

Samples	*E*_corr_ (mV)	*I*_corr_ (mA)	Corrosion rate (mm y^−1^)
Cu-coated PPC 1	−51.683	6.831	0.083
Cu-coated PPC 2	−56.007	6.157	0.075
Cu-coated PPC 3	−56.832	7.423	0.091

**Table:** Corrosion factors determined from the test in 0.01 M H_2_SO_4_

Samples	*E*_corr_ (mV)	*I*_corr_ (mA)	Corrosion rate (mm y^−1^)
Cu-coated PPC 1	−73.787	18.060	0.312
Cu-coated PPC 2	−74.991	14.361	0.248
Cu-coated PPC 3	−60.761	13.115	0.227

n an acid scenario, copper-coated PPCs were corroded with the corrosion rate range of 0.227–0.312 mm y^−1^. It is worth noting that the copper-coated PPC 3 (0.227 mm y^−1^) showed the best corrosion resistance among other coating materials. The corrosion potential, corrosion current, and corrosion rate values of the coated specimens tested in sulfuric acid (pH = 2) enabled higher corrosion current values in comparison with the test in methanol solution. The results indicated that copper was easily corroded in the fuel cell condition, so coating a copper material on the outermost layer is not proper. The multilayer coating with a high corrosion resistant material on the outermost layer may be required.^21^ As mentioned before, the copper coating on polymer composite BPs is still interesting, because composites are easy to be coated with copper, copper layer is strong, and production cost is inexpensive.

**Fig. 18:**
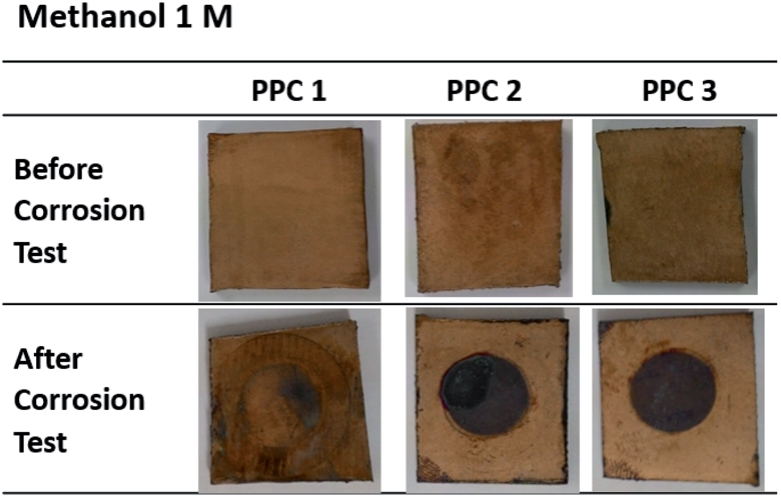
Surface changes of copper-coated PPCs before and after corrosion tests in 1.00 M CH_3_OH.

**Fig. 19:**
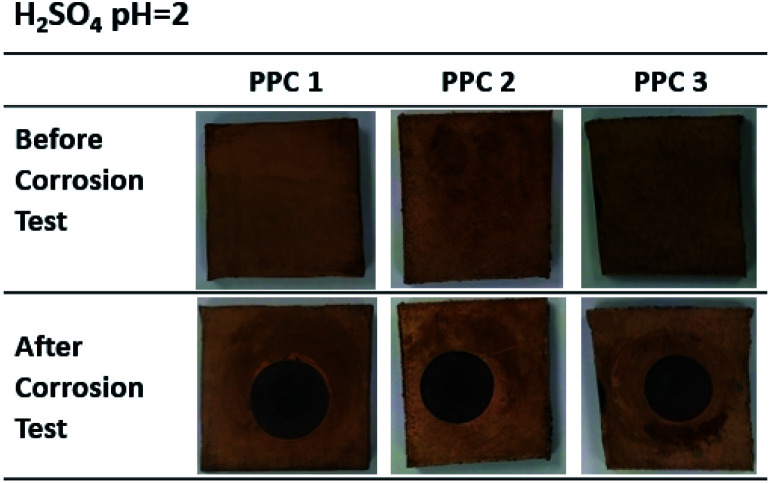
Surface changes of copper-coated PPCs before and after corrosion tests in 0.01 M H_2_SO_4_.

Fig. 18 and 19 exhibit a surface character of coated samples before and after corrosion tests. After the test in methanol solution, copper layers of coated PPC 1 and PPC 2 surfaces were peeled off as seen black circles on their surfaces. In the case of sulfuric acid solution, the black circles can be remarked on all sample surfaces. Surface features indicated that some copper had peeled off and the copper colour slightly turned to be green. The change in colour relates to a chemical change, so it implies that peeling occurs together with corrosion. Consequently, cathodic delamination technique *via* electrochemical impedance spectroscopy (EIS) was applied to confirm that the copper layer on composite BP was peeled.

**Fig. 20:**
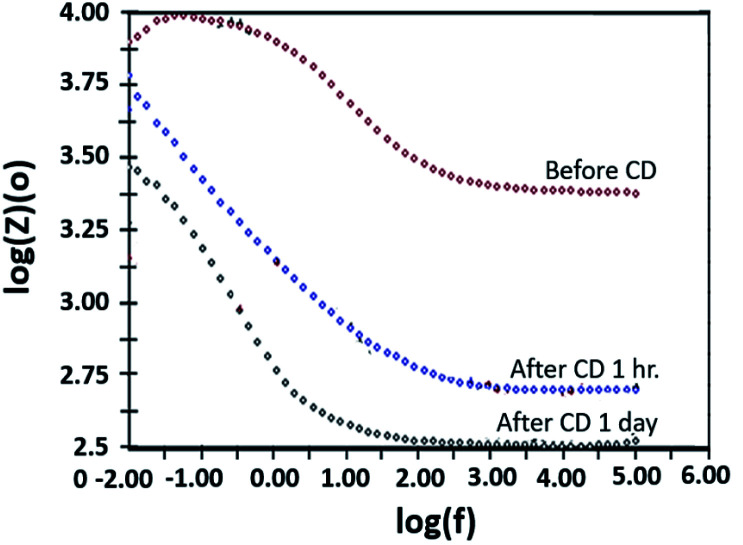
Bode plot copper-coated PPC 3 from cathodic delamination technique.

**Fig. 21:**
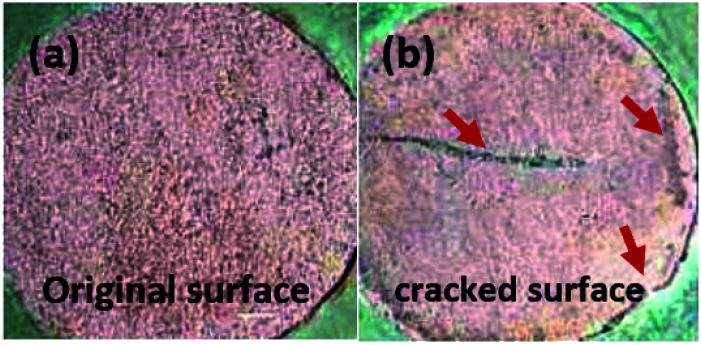
The surface features of samples; (a) original surface and (b) cracked surface, were used for cathodic delamination process.

The copper-coated PPC 3 was chosen to be a sample for the EIS test, since the DMFC assembled with BP made from copper-coated PPC 3 was comparable in performance to a commercial BP made from a polymer composite. This test was a preliminary experiment to be guideline for studying surface corrosion and delamination. The EIS test, where a copper-coated PPC 3 was immersed in a methanol solution at various durations of time (1 hour and 1 day). The Bode plot in Fig. 20 reported the relationship between the total resistance of the system and the frequency. The results indicate that after going through the cathodic delamination process for 1 hour, the total resistance was greatly reduced because the copper layer was dislodged. After one day of the cathodic delamination process, the methanol solution penetrated the seams of the composite plate resulting in more peeling leading to an even higher decrease in the resistance. The total resistance from 1 hour was not much different from that of a one day process. At the lowest frequency, cathodic delamination slightly reduced the total resistance, since the surfaces were partially cracked. These partially cracked surfaces caused an increase in surface area of copper layer leading to surface electrical conductivity elevation (Fig. 21). A reduction in the total resistance occurred after the methanol solution diffused into the copper layer, and then the layer was partially delaminated. Small copper particles were removed from the composite surfaces and dispersed in the solution. Electrically conductive paths created by the copper particles resulted in the resistance of the solution. Thus, the increase in total resistance of the CD test system was decreased.

Typically long-term test is mandatory to observed fuel cell durability. As usually, automotive applications require more than 5000 hours of fuel cell lifetime, while residential applications need longer lifetime than 20 000 hours in order to be used in different environmental conditions.^55^ The durability will be tested in the future after developing metal coating performance regarding to solving the corrosion and adhesion issues. The mentioned durability tests may be cathodic delamination both acid and methanol and EIS analysis during cell operation.

## 4. Conclusions

A prototype of copper-coated composite bipolar plates was produced to demonstrate the method of reducing surface resistance in a direct methanol fuel cell. The coating procedure that is based on an electroless deposition technique was used to demonstrate an easier process of manufacturing and reasonably lower costs in potential production. The success in coating copper on surface of polypropylene/carbon filler composite bipolar plates was confirmed by physicochemical characterizations. However, surface corrosion and delamination of copper layer were issues needed to be solved. The following conclusions were extracted from this research:

(1) Plasma treatment enhanced adhesion performance, and the optimum plasma treatment time was 30 seconds.

(2) Physicochemical characterizations *via* XPS, FTIR-ATR, and contact angle determination asserted that the plasma treatment, silanization, and copper electroless deposition were successful.

(3) The 0.06 mol L^−1^ of Cu^2+^ concentration was prepared to coat PPC plates at 40.0 °C of operating temperature.

(4) The electrical conductivity of the surface of all copper-coated BPs was in the range of 275 to 399 S cm^−1^ which was higher than the requirement for the BP application.

(5) The copper-coated PPC 3 bipolar plate was as effective as the commercial epoxy composite bipolar plate. The DMFC assembled with copper-coated PPC 3 bipolar plates delivered 23.18 mW cm^−2^ of power density and 65% of efficiency.

(6) Copper was corroded in the fuel cell condition, so coating a copper material on the outermost layer is not proper. A multilayer coating technique may solve this corrosive problem.

(7) Copper delamination caused by methanol solution affected the durability of DMFC, so the interface adhesion performance required improvement.

## Conflicts of interest

There are no conflicts of interest to declare

## Supplementary Material
